# Environmental Applications of Mass Spectrometry for Emerging Contaminants

**DOI:** 10.3390/molecules30020364

**Published:** 2025-01-17

**Authors:** Anil Kumar Meher, Akli Zarouri

**Affiliations:** Department of Bioproducts and Biosystems Engineering, University of Minnesota, St. Paul, MN 55108, USA; azarouri@umn.edu

**Keywords:** emerging contaminants, mass spectrometry, LC-MS, GC-MS, PTR-MS, data analysis

## Abstract

Emerging contaminants (ECs), encompassing pharmaceuticals, personal care products, pesticides, and industrial chemicals, represent a growing threat to ecosystems and human health due to their persistence, bioaccumulation potential, and often-unknown toxicological profiles. Addressing these challenges necessitates advanced analytical tools capable of detecting and quantifying trace levels of ECs in complex environmental matrices. This review highlights the pivotal role of mass spectrometry (MS) in monitoring ECs, emphasizing its high sensitivity, specificity, and versatility across various techniques such as Gas Chromatography-Mass Spectrometry (GC-MS), Liquid Chromatography-Mass Spectrometry (LC-MS), and High-Resolution Mass Spectrometry (HR-MS). The application of MS has facilitated the real-time detection of volatile organic compounds, the comprehensive non-targeted screening of unknown contaminants, and accurate quantification in diverse matrices including water, soil, and air. Despite its effectiveness, challenges such as matrix interferences, a lack of standardized methodologies, and limited spectral libraries persist. However, recent advancements, including hybrid MS systems and the integration of artificial intelligence (AI), are paving the way for more efficient environmental monitoring and predictive modeling of contaminant behavior. Continued innovation in MS technologies and collaborative efforts are essential to overcome existing challenges and ensure sustainable solutions for mitigating the risks associated with emerging contaminants.

## 1. Introduction

**Figure 1:**
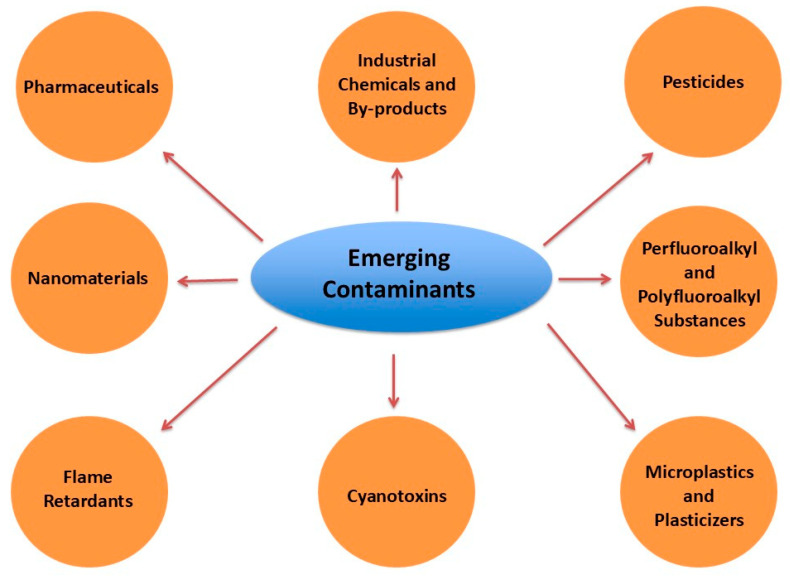
Categories of emerging contaminants.

Emerging contaminants (ECs), or contaminants of emerging concern (CECs), include various chemical substances with potential risks to human health and ecosystems due to their persistence and toxicity [1]. These substances have been identified in the environment and consist of pharmaceuticals [2], personal care products [3], endocrine disruptors [4], and industrial chemicals [5] are summarized in Figure 1. Despite their presence, they largely remain unregulated or inadequately monitored. Many of these contaminants enter ecosystems through wastewater, agricultural runoff, or industrial emissions and landfill leachate [6], posing potential risks to both ecosystems and human health. The persistence and potential bioaccumulation of ECs, combined with their often-unknown toxicological profiles, can disrupt natural habitats, alter biodiversity, and introduce health risks when they infiltrate drinking water sources [7].

ECs are constantly evolving as substances initially identified become recognized as environmental threats with advancing scientific insights. This category includes both newly synthesized chemicals and “legacy” contaminants which are familiar substances like lead and arsenic. Their effects and environmental behaviors are now being reconsidered as new data surfaces. Pollutants such as cyanotoxins from algal blooms and by-products from water treatment processes illustrate the extensive and unpredictable range of these contaminants. The complex, interconnected effects of these pollutants highlight the necessity for multidisciplinary approaches to thoroughly evaluate their impacts on human health, ecosystems, and natural resources. The criteria for classifying a substance as an emerging contaminant hinge on several key factors, which underscore their potential risks to ecosystems and human health. First, persistence in the environment is a fundamental trait; ECs tend to resist natural degradation processes, allowing them to remain active and accumulate over extended periods [8]. This persistence makes them difficult to manage through conventional treatment or remediation processes, increasing their chances of widespread distribution. Another important factor is bioaccumulation, as many ECs are capable of concentrating within biological systems, potentially transferring from one organism to another across trophic levels [9]. This can lead to long-term ecological impacts, as these contaminants travel up the food chain, posing threats to wildlife and, eventually, humans who consume contaminated species.

Toxicity is also a significant criterion, although toxicological data on many ECs remain incomplete. Despite this limitation, some ECs have been linked to harmful effects on various organisms, particularly through endocrine and reproductive disruptions. For example, pharmaceuticals and personal care products that act as endocrine disruptors can alter hormonal balances in aquatic species, leading to population-level effects [10]. Finally, ubiquity and detection are key considerations. ECs are increasingly detected in a wide range of environments, from urban and rural water systems to remote ecosystems like Arctic waters and even atmospheric layers. Their ability to spread across diverse habitats further emphasizes their potential for far-reaching impacts. Together, these criteria highlight the complexity of managing ECs and the need for enhanced monitoring, regulation, and public awareness to address their environmental and health implications effectively [11].

In tackling ECs, researchers and policymakers must navigate challenges in prioritization and risk assessment. Limited toxicological data, along with varying persistence and degradation rates, complicate efforts to establish safe exposure thresholds and environmental guidelines. The constantly evolving landscape of these contaminants calls for adaptive management strategies and enhanced funding for research. Proactive monitoring and advanced treatment technologies are essential to mitigate the risks associated with these pollutants, yet resource constraints and gaps in public awareness continue to hinder widespread implementation.

The detection and quantification of these contaminants are essential for environmental safety, as many of them exhibit toxic properties even at low concentrations. Increased detection efforts have highlighted the widespread distribution of ECs across different environmental media, including soil, water, and air. The environmental behavior of these contaminants is complex and often varies with the chemical composition, posing challenges to effective environmental management. Their potential to affect various ecological and human health systems has prompted regulatory agencies to monitor their prevalence and influence, especially in sensitive ecosystems like aquatic environments [7].

## 2. Mass Spectrometry for EC Analysis

MS has emerged as a critical analytical tool in identifying and quantifying ECs in complex environmental samples. MS has become an invaluable analytical tool in detecting and quantifying ECs within environmental samples. Its high sensitivity, selectivity, and adaptability make it especially suitable for analyzing trace contaminants across various environmental media, such as water, soil, and air. Several MS techniques stand out for environmental applications, each offering unique advantages based on the characteristics of the contaminants. Gas Chromatography-Mass Spectrometry (GC-MS) is commonly used for analyzing volatile and semi-volatile organic compounds, such as industrial chemicals and certain pesticides. By separating compounds based on their volatility before they reach the MS detector, GC-MS enables precise identification and quantification, making it a preferred method for monitoring the air quality and soil contamination.

LC-MS, in contrast, is well-suited for non-volatile and thermally unstable compounds, such as pharmaceuticals and personal care products. LC-MS separates analytes based on their solubility in different solvents, allowing it to handle the complex matrix of environmental samples like wastewater. This technique is highly effective for monitoring ECs in aquatic environments, where such contaminants are often found in low concentrations.

Proton Transfer Reaction Mass Spectrometry (PTR-MS) is a real-time MS technique primarily used for detecting volatile organic compounds (VOCs) directly in the air without the need for pre-separation. This high-throughput capability makes PTR-MS suitable for continuous environmental monitoring, especially for compounds that rapidly disperse or change concentrations, such as VOCs from industrial emissions.

Lastly, Selected Ion Flow Tube Mass Spectrometry (SIFT-MS) [12] is another direct MS method that provides the real-time detection of trace gases in complex samples. Like PTR-MS, SIFT-MS does not require pre-separation, but instead uses controlled chemical ionization to achieve high sensitivity and specificity. This method is effective for detecting reactive and labile compounds in ambient air, making it particularly useful for monitoring pollutants with short atmospheric lifetimes.

The selection of GC-MS, LC-MS, PTR-MS, and SIFT-MS for discussion in this section reflects the diverse analytical needs in environmental monitoring and the unique strengths of each technique. GC-MS and LC-MS are well-established and widely used methods for the analysis of semi-volatile and non-volatile compounds, respectively, offering high sensitivity and specificity across various environmental matrices such as air, water, and soil. Their versatility makes them indispensable for both targeted and non-targeted analyses. PTR-MS and SIFT-MS, on the other hand, are specialized techniques for the real-time analysis of VOCs. Although they serve similar functions, they employ distinct ionization mechanisms: PTR-MS relies on proton transfer reactions, enabling the high-throughput monitoring of VOCs, while SIFT-MS utilizes soft chemical ionization with reagent ions (H_3_O^+^, NO^+^, O_2_^+^), providing greater control over ion–molecule reactions and enhancing the specificity for certain compounds. By presenting these techniques together, this section highlights a spectrum of analytical approaches, from conventional chromatography-based methods to cutting-edge direct analysis techniques, demonstrating their complementary roles in addressing the challenges of environmental contaminant analysis.

These MS techniques collectively offer a robust toolkit for detecting diverse ECs, providing crucial insights into their distribution, behavior, and potential risks in the environment. Their application allows scientists to track contamination patterns, identify sources, and assess ecological impacts, informing both remediation efforts and regulatory policies.

MS techniques offer high sensitivity and specificity, making them ideal for detecting trace amounts of ECs. The precision and accuracy of MS enable environmental scientists to monitor contamination levels accurately, trace pollution sources, and evaluate potential ecological impacts. This review focuses on discussing the various applications of MS in detecting and studying ECs, with an emphasis on recent advancements and challenges. The primary objective of this review is to provide an in-depth analysis of the role of MS in the environmental monitoring of ECs, exploring its methodologies, applications, and limitations. By reviewing key studies and recent developments, this article aims to contribute to the growing body of knowledge on ECs and support the development of effective regulatory and mitigation strategies for environmental safety.

## 3. Factors Influencing Selection of Mass Spectrometry Method in EC Studies

**Figure 2:**
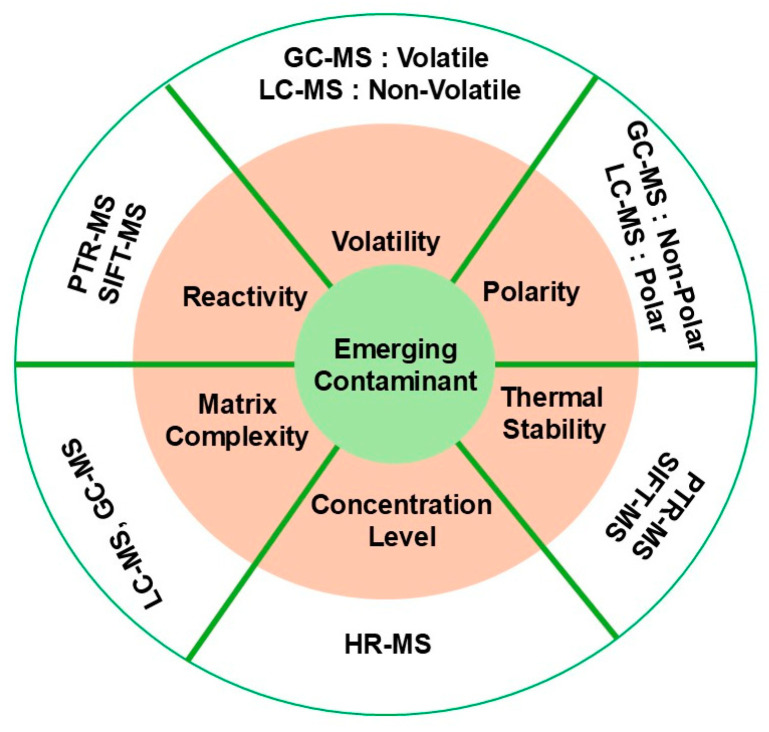
Parameters influencing the selection of MS instruments for analysis of ECs categorized by factors such as volatility, polarity, reactivity, and matrix complexity.

The selection of an appropriate MS technique for detecting ECs depends on several key characteristics of the contaminants, such as their volatility, polarity, thermal stability, concentration levels, and the complexity of the matrix in which they are found. Each MS technique has distinct advantages based on these factors, so making the right choice is essential for accurate, sensitive detection (Figure 2).

### 3.1. Volatility

The volatility of a contaminant plays a significant role in choosing between GC-MS and LC-MS. GC-MS is ideal for volatile and semi-volatile compounds that can be vaporized without decomposition, like benzene, toluene, and some pesticides, which are commonly found in industrial and agricultural settings [13]. LC-MS, however, is preferred for non-volatile or thermally sensitive compounds, such as antibiotics and steroid hormones, which would degrade if vaporized [14].

### 3.2. Polarity

The polarity of ECs influences their separation and detection efficiency in chromatography [15]. LC-MS is particularly effective for polar and ionic compounds like pharmaceutical residues (e.g., acetaminophen) and certain personal care products as it separates these substances in a liquid phase. In contrast, GC-MS is well-suited for non-polar or moderately polar contaminants, like polycyclic aromatic hydrocarbons (PAHs) and certain plasticizers, which are more stable in a gaseous phase. For instance, Ostadgholami et al. highlight that GC-MS is an efficient analytical technique for multiple contaminants characterized by thermal stability, lower polarity, and higher volatility, making it ideal for the analysis of PAHs and similar compounds [16]. This capability is essential for environmental monitoring, where the presence of such contaminants poses significant ecological risks. Similarly, Vandergrift et al. emphasize the use of GC-MS for the quantitation of PAHs in soil samples, noting that this method is frequently employed due to its effectiveness in detecting these carcinogenic compounds [17]. The authors point out that GC-MS, often coupled with initial sample extraction and cleanup steps, is a standard approach for analyzing PAHs in various environmental matrices. In the context of environmental monitoring, the study by Badawy et al. reviewed modern principles and applications of solid-phase extraction (SPE) techniques in chromatographic analysis, which are often coupled with GC-MS for the analysis of plasticizers in environmental samples [18]. The authors highlighted the effectiveness of these techniques in isolating plasticizers from complex environmental matrices, thereby enhancing the overall analytical performance.

Overall, the literature supports the assertion that GC-MS is well-suited for the analysis of non-polar or moderately polar contaminants, particularly polycyclic aromatic hydrocarbons and certain plasticizers, due to its efficiency in handling compounds that are stable in the gaseous phase.

### 3.3. Thermal Stability

For contaminants that are thermally unstable, such as certain pesticides like carbamates or chemicals in personal care products, LC-MS or techniques that do not involve sample heating, like Selected Ion Flow Tube Mass Spectrometry (SIFT-MS) [19] or PTR-MS [20], are optimal. These techniques avoid the high temperatures in GC-MS, preserving the integrity of compounds that would otherwise degrade. The research conducted by Hu and colleagues focused on the thermal degradation of phenolic carbamates in dynamic polyurethane networks. They observed that decomposition occurs between 240 and 260 °C, emphasizing the thermal instability of these compounds. Using LC-MS, the study effectively identified the degradation products, demonstrating the technique’s ability to analyze thermally unstable species without causing further degradation. Zamani et al. explored the thermal decomposition of carbamates at temperatures exceeding 160 °C. They highlighted the limitations of conventional analytical methods, such as gas chromatography, in studying these compounds due to their high reactivity and thermal instability. To overcome these challenges, the researchers utilized LC-MS to investigate the degradation products, showcasing the technique’s suitability for analyzing thermally unstable substances [21]. Jiang et al., in their research on ethanol-based disinfectant sprays, highlighted the rapid changes in the chemical composition of indoor air. They utilized LC-MS to identify thermally unstable compounds emitted from these disinfectants, demonstrating the technique’s effectiveness in capturing transient species that would decompose under heat [22]. Ghislain et al. [23] explored the chemical ionization of organophosphate esters, which are emerging environmental contaminants. Their study demonstrated that SIFT-MS is particularly suitable for analyzing these compounds in air due to its direct analytical capabilities, which require minimal sample preparation and avoid thermal degradation. This method allows for the real-time detection of semi-volatile organic compounds, making it a valuable tool for environmental monitoring. Yuan et al. discussed the photochemical transformation products of environmental organic contaminants in aquatic systems [24]. While the focus was on predicting transformation products, the study underscores the relevance of using soft ionization techniques like PTR-MS to analyze the resulting compounds without thermal degradation, thereby providing accurate assessments of environmental contaminants.

### 3.4. Concentration Levels and Sensitivity Needs

Some ECs exist in trace amounts, requiring techniques with high sensitivity. High-Resolution Mass Spectrometry (HR-MS) and Tandem MS (MS/MS) offer precise quantification even at low concentrations, which is essential for contaminants like endocrine-disrupting compounds (e.g., bisphenol A) in aquatic systems [25]. Yang et al. in 2022 [26] developed a precolumn derivatization method for the determination of perfluorocarboxylic acids (PFCAs) in catalytic degradation solutions. They noted that while their LC-UV method is not recommended for trace amounts of PFCAs in environmental samples, LC-MS is more sensitive and advantageous for excluding false-positive results, making it suitable for low-concentration measurements. The study by Barret et al. highlighted class-specific temporal trends of poly- and perfluoroalkyl substances in beluga whales through the application of suspect and non-target screening. The authors underscored the critical role of sensitive analytical techniques, such as high-resolution mass spectrometry (HR-MS), in monitoring trace levels of these contaminants in biological samples [27].

For the continuous monitoring of VOCs in the air, SIFT-MS and PTR-MS are advantageous as they enable real-time, high-sensitivity detection.

### 3.5. Matrix Complexity

The environmental matrix—whether water, soil, air, or biota—significantly impacts MS technique selection. For instance, LC-MS, often combined with SPE, is ideal for detecting pharmaceuticals and pesticides in water. Pereira et al. demonstrated the application of LC-MS/MS coupled with SPE for the detection of trace pharmaceuticals in environmental water matrices, achieving detection limits as low as 1.13 to 5.45 ng L^−1^ [28]. The use of SPE allowed for the effective pre-concentration and cleanup of complex water samples, enhancing the sensitivity and precision of the analysis. Their work highlights the ability of this combined method to analyze a wide range of pharmaceuticals, including antibiotics, SSRIs, NSAIDs, and hormones in drinking water, tap water, and groundwater samples. By employing matrix-matched calibration and multiple reaction monitoring (MRM), they minimized the matrix effects, further improving the reliability of the quantitative analysis. This study underscores the robustness of mass spectrometry, in conjunction with SPE, in assessing pharmaceutical contamination and its environmental implications [28].

GC-MS, on the other hand, is highly effective for analyzing air samples for industrial pollutants and soil samples for semi-volatile compounds like PAHs. Kamalova et al. explored the application of advanced mass spectrometry in conjunction with SPE for the efficient determination of polycyclic aromatic hydrocarbons (PAHs) in soil matrices [29]. By integrating SPE with GC-MS, the study achieved high sensitivity and specificity in detecting PAHs, even at trace levels. The use of SPE for the pre-concentration and matrix cleanup significantly improved the extraction of heavier PAHs such as pyrene and chrysene, which are typically challenging to isolate due to their low volatility and high molecular weight. This method underscores the importance of coupling robust sample preparation techniques like SPE with GC-MS to ensure the accurate quantification of complex contaminants, providing valuable insights into the environmental pollution assessment and remediation strategies [29]. For gaseous matrices, PTR-MS [30] and SIFT-MS [31] are particularly useful, providing real-time analysis without extensive sample preparation. Recently, Langford et al. reviewed the adoption of selected ion flow tube mass spectrometry (SIFT-MS) for environmental monitoring, emphasizing its real-time capabilities in detecting VOCs [32]. They highlighted its broad applicability, from emission source characterization to ambient monitoring and pollution mapping. Unlike conventional GC-based methods, SIFT-MS eliminates the need for extensive sample preparation or chromatographic separation, enabling faster and more flexible analysis across diverse environmental settings. This review underscores the potential of SIFT-MS as a complementary tool to regulatory techniques, providing rapid and accurate insights critical for addressing dynamic environmental challenges.

### 3.6. Reactivity and Chemical Composition

For highly reactive or labile compounds, rapid techniques like PTR-MS or SIFT-MS, which avoid lengthy sample preparation, are suitable. These methods enable the fast, real-time detection of reactive VOCs from industrial emissions, such as formaldehyde and acetaldehyde, which can change their composition over time if not analyzed immediately. The use of PTR-MS for the detection of formaldehyde and acetaldehyde has been well-documented. Wu et al. noted that PTR-MS is capable of detecting a wide range of oxygenated VOCs, including formaldehyde and acetaldehyde, with high sensitivity and rapid response times [33]. SIFT-MS is known for its ability to perform a direct analysis of VOCs without the need for extensive sample preparation, which is a significant advantage over traditional methods such as GC-MS. Perkins et al. emphasize that SIFT-MS can analyze the headspace directly, allowing for quicker results compared to methods that require chromatographic separation [34]. This capability is particularly beneficial in scenarios where a time-sensitive analysis is crucial, such as in the monitoring of emissions from industrial processes. Moreover, studies have shown that SIFT-MS provides higher detection sensitivity and is less affected by environmental factors like humidity, which can interfere with the measurement of reactive VOCs [35]. Choi’s research highlights that SIFT-MS demonstrated slightly higher detection amounts of phytoncides compared to GC-MS, reinforcing its effectiveness in real-time monitoring applications [35]. This is particularly relevant in industrial settings where VOC emissions can fluctuate rapidly, necessitating a method that can keep pace with these changes.

By carefully considering these characteristics, researchers can select the MS technique that provides optimal sensitivity, specificity, and reliability for detecting and quantifying ECs in diverse environmental samples. This approach ensures precise data collection on EC prevalence and their environmental impact, guiding effective monitoring and regulatory decisions.

## 4. Role of Tandem Mass Spectrometry in Environmental Analysis

Tandem mass spectrometry (MS/MS) plays a pivotal role in environmental analysis, providing high sensitivity and specificity for the identification and quantification of trace-level contaminants [36]. This capability is particularly important for analyzing complex environmental matrices such as water, soil, and air [37]. Many LC-MS and GC-MS techniques incorporate tandem mass spectrometry functionality, which enhances their analytical capabilities, particularly in the analysis of environmental samples. The integration of MS/MS allows for improved specificity and sensitivity, making these methods essential for detecting trace contaminants in complex matrices. For instance, Jamur discusses the importance of developing analytical methodologies for the determination of metronidazole and trimethoprim in environmental samples, emphasizing the role of mass spectrometry in accurately identifying these compounds [38]. The study highlights that MS/MS techniques are particularly effective for analyzing low-concentration contaminants in environmental matrices, showcasing their utility in environmental monitoring. Furthermore, the work by Justen et al. focuses on the quantification of disinfection byproducts (DBPs) in drinking water using liquid–liquid extraction followed by gas chromatography-tandem mass spectrometry (LLE-GC-MS/MS) [39]. The authors emphasize that the MS/MS capabilities allow for the detection of DBPs at low concentrations, which is crucial for assessing potential health risks associated with the water quality.

In environmental applications, LC-MS/MS is widely used for detecting non-volatile and thermally unstable contaminants such as pharmaceuticals [40], personal care products [41], and endocrine-disrupting chemicals [42]. For example, LC-MS/MS enables the detection of bisphenol A [43], triclosan [44], and other contaminants at trace levels in complex water matrices, including wastewater and river water [45]. Similarly, GC-MS/MS is highly effective for volatile and semi-volatile organic compounds, such as pesticides [46,47], polycyclic aromatic hydrocarbons (PAHs), and persistent organic pollutants (POPs) [48], providing precise quantification even in challenging matrices like air and soil.

Tandem MS is particularly powerful for non-targeted analyses, where the structural elucidation of unknown compounds is critical. Fragmentation patterns generated in MS/MS provide insights into the chemical structures of unknown contaminants and their transformation products, which are often formed during natural processes or treatment interventions. This is exemplified in studies identifying byproducts of water treatment processes, where tandem MS has been instrumental in characterizing the persistence and toxicity of ECs and their derivatives.

Additionally, MS/MS is integral to biomonitoring studies, where it quantifies bioaccumulated contaminants in biological samples such as fish, birds, and plants. These studies have highlighted the ecological risks associated with pollutants like polychlorinated biphenyls (PCBs) and per- and polyfluoroalkyl substances (PFASs). The high specificity of MS/MS ensures the reliable quantification of these compounds, even in matrices with high levels of background interference.

While the inherent use of MS/MS in LC-MS and GC-MS techniques has broadened their applicability, challenges remain. These include matrix effects, ion suppression, and the need for standardized protocols across various environmental matrices. However, continuous advancements in tandem MS instrumentation, including the development of triple, quadrupole, and hybrid systems, have significantly enhanced the analytical robustness. These improvements allow for more accurate quantification, better structural elucidation, and faster analysis, addressing the evolving needs of environmental monitoring.

## 5. Sample Preparation and Extraction Methods for EC Analysis

Preparing samples and extracting compounds are crucial steps in identifying ECs, especially when using advanced techniques to screen for known and unknown substances. Since environmental, biological, and food samples often come with complex mixtures, these steps must be carefully tailored to ensure accurate results while minimizing interference from other materials in the sample. Common methods like solvent extraction and SPE are widely used, with SPE being particularly effective for isolating contaminants from liquids [49]. To tackle the diversity of chemicals present, researchers often use a combination of different SPE sorbents or sequential solvent extractions, aiming to capture a wide range of substances. Materials like Oasis HLB and C [18] are frequently chosen for their ability to handle challenging samples, though no single approach is perfect for every situation [50,51]. Dispersive micro SPE (DMSPE) is a promising approach that simplifies the SPE process by using sorbent mixtures for the direct in situ preconcentration of analytes, while fast on-line SPE introduces automation, improved repeatability, and sub-ng L^−1^ detection limits [52].

Advanced techniques, such as gel permeation chromatography (GPC), help remove unwanted lipids and other impurities, though they can sometimes cause the loss of important compounds [53]. Recently, innovative solutions like ionic liquids [54], nanomaterials [55], and molecularly imprinted polymers [56] have started to make a difference by improving the precision and efficiency of extractions. A recent review by Sereshti et al. provides a comprehensive overview of nanosorbent-based solid-phase microextraction (SPME) techniques for monitoring emerging organic contaminants (EOCs) in water and wastewater samples. The authors discuss the preparation, properties, advantages, and limitations of various nanosorbents used in SPME applications, including carbon-based materials like graphene and carbon nanotubes, as well as magnetic nanosorbents and metal–organic frameworks. They highlight that the selection of sorbent material is crucial for the efficiency of SPME, as it directly affects the extraction performance for target pollutants [57].

In some cases, the direct injection of samples into analytical instruments is an option, but it often struggles to detect low-concentration contaminants or deal with complex sample types. The field still faces challenges, including the lack of standardized methods and the difficulty of validating results without established reference materials. Despite these obstacles, researchers continue to develop and refine techniques, making it possible to uncover and better understand the hidden chemicals that may impact our environment and health. For instance, the use of emerging techniques such as Extractive Electrospray Ionization Mass Spectrometry (EESI) [58] has been explored by Wu, who describes how this method allows for the direct ionization of analytes under ambient conditions. This capability significantly streamlines the sample preparation phase, facilitating real-time analyses of complex samples, including those from environmental contexts. Also Nazdrajić et al. discuss the coupling of Solid-Phase Microextraction (SPME) [59] directly to mass spectrometry via an improved microfluidic open interface, which facilitates high-throughput determinations. This method minimizes the matrix effects and reduces the risk of instrument contamination, making it particularly suitable for analyzing complex samples containing ECs. Additionally, the authors emphasize that SPME offers matrix-compatible coatings that enhance analyte enrichment, thereby improving detection limits and the overall analytical performance. Godfrey et al. demonstrated the significant potential of the QuEChERS [60] (Quick, Easy, Cheap, Effective, Rugged, and Safe) method as an innovative approach for environmental analysis. They adapted the QuEChERS method, originally developed for pesticide extraction in food, to various environmental matrices such as wastewater effluent, sludge, and biota. This adaptation reduced extraction times from hours to just 20 min per sample while requiring minimal resources. Additionally, Godfrey et al. highlighted the method’s flexibility for high-throughput and onsite analyses, as well as its potential for automation, making it a highly effective tool for pollution monitoring and regulatory applications. In summary, the landscape of sample preparation and extraction methods for analyzing ECs by mass spectrometry is evolving with the introduction of innovative techniques that enhance the efficiency, sensitivity, and throughput. The integration of methods like SPME, QuEChERS, and EESI-MS reflects a growing trend toward simplifying the analytical process while maintaining high standards of accuracy and reliability.

## 6. Environmental Matrices and Challenges in MS Analysis

Analyzing ECs in diverse environmental matrices presents significant challenges, particularly when employing MS techniques. Environmental samples such as water, sediments, soils, and air are complex and heterogeneous, often containing a multitude of organic and inorganic substances that can interfere with the detection and quantification of ECs. For instance, sediments may harbor various polar pesticides that are difficult to extract and analyze due to their low hydrophobicity and potential degradation during sample preparation [61]. The presence of natural organic matter, humic substances, and other co-contaminants can suppress or enhance ionization in MS, leading to inaccurate quantification. Moreover, the transformation products of ECs, formed through biological or chemical degradation, add another layer of complexity. High-resolution mass spectrometry (HRMS) has emerged as a powerful tool to identify these transformation products, offering high mass accuracy and the ability to perform non-target screening [62]. Non-target screening captures a broad range of compounds, including those previously unknown, by using full-scan mass spectra. This approach allows researchers to revisit data as new contaminants or transformation products are identified [63]. With the precision of high-resolution mass spectrometry (HRMS) and tools like suspect screening and chemometric analysis, scientists can uncover hidden patterns in complex samples, gaining deeper insights into contamination and discovering pollutants we might not have recognized before. By offering insights into both known and unknown ECs, non-target screening provides a more comprehensive understanding of contamination patterns, facilitating the discovery of previously unrecognized contaminants and their transformation products. This addition improves the clarity and depth of the discussion on the value of HRMS in EC analysis [64].

Recently, Nguyen et al. addressed the challenges of analyzing multi-class ECs in complex environmental matrices like soil and sediment, emphasizing the need for robust methodologies to extract trace-level contaminants while minimizing interference from organic matter. The study optimized a QuEChERS-based extraction method coupled with UPLC-MS/MS for 90 emerging organic contaminants, including pesticides, pharmaceuticals, and PFASs, achieving recoveries between 70% and 120% with low matrix effects. The use of citrate buffer and 1–2% formic acid significantly improved recoveries for diverse chemical classes, highlighting the importance of pH optimization in enhancing the extraction efficiency [65].

However, the lack of standardized protocols and comprehensive spectral libraries for these ECs and their transformation products hampers the identification process. Additionally, the continuous introduction of new chemicals into the environment necessitates constant updates to analytical methods and databases. Addressing these challenges requires the development of robust sample preparation techniques, advanced MS methodologies, and comprehensive databases to ensure the accurate detection and assessment of ECs across various environmental matrices. The continuous introduction of new chemicals into the environment exacerbates these difficulties. For example, novel flame retardants or pesticides introduced as replacements for banned substances may lack comprehensive toxicological and spectral data, leaving a gap in the detection capabilities [66]. This dynamic chemical landscape necessitates constant updates to analytical methods and databases to keep pace with the evolving range of contaminants.

To address these challenges, several ongoing efforts aim to standardize methods and create comprehensive libraries. For example, the U.S. Environmental Protection Agency (EPA) has been actively developing non-targeted analysis (NTA) protocols and the CompTox Chemicals Dashboard, which aggregates chemical structures, experimental data, and predicted properties for thousands of chemicals [67,68]. Additionally, the European NORMAN Network is fostering collaboration among laboratories to harmonize workflows and develop shared spectral libraries for ECs and their metabolites [69].

## 7. Applications of MS in Monitoring ECs

MS has become indispensable in monitoring ECs across diverse environments, offering unmatched sensitivity and specificity. In aquatic systems, MS techniques like LC-MS/MS and HRMS are widely used to detect pharmaceuticals, personal care products, and other micropollutants in water bodies, even at trace levels [70]. Nazar et al. utilized GC-MS/MS and LC-MS/MS to analyze 345 micropollutants, including pesticides and endocrine disruptors, in fish from the Cochin estuary, highlighting the bioaccumulation of these contaminants and their associated health risks [71]. Similarly, atmospheric monitoring benefits from MS techniques like PTR-MS, which detect industrial pollutants such as VOCs in real-time. For soil and sediments, MS-based methods effectively trace pesticide residues and persistent organic pollutants, aiding environmental risk assessments. In biological matrices, MS applications extend to biomonitoring ECs in wildlife, as demonstrated by the analysis of fish tissues to identify endocrine disruptors and polyaromatic hydrocarbons. These studies underscore the versatility of MS in providing robust, high-sensitivity detection across environmental and biological contexts. Atmospheric monitoring benefits from MS methods such as PTR-MS and DART-MS facilitate the real-time analysis of VOCs and industrial pollutants, providing insights into air quality and pollutant dispersion. Soil and sediment analysis relies on GC-MS and LC-MS to trace pesticide residues and persistent organic pollutants, revealing contamination pathways and environmental persistence. In aquatic systems, MS methods like LC-MS/MS and GC-MS/MS enable the detection of trace levels of pharmaceuticals, pesticides, and other ECs in water and sediments, as highlighted by Peris and Eljarrat. Their work underscores the importance of multi-residue analysis for tracking contaminants like oxadiazon, which pose significant risks to aquatic organisms [46]. For biological samples, MS-based biomonitoring identifies ECs and their metabolites in wildlife, such as polar bears and amphibians, elucidating bioaccumulation and ecological risks. Jasrotia et al. emphasize the widespread impact of endocrine-disrupting chemicals (EDCs) on aquatic ecosystems, highlighting their detrimental effects on wildlife and human health [72]. These chemicals, originating from industrial, agricultural, and pharmaceutical sources, disrupt the hormonal balance and affect reproduction, development, and metabolism in aquatic organisms. The study reveals the bioaccumulation of EDCs through food webs, leading to significant ecological and health risks. Common contaminants include heavy metals, flame retardants, and pesticides, which interfere with endocrine signaling pathways. Efforts to mitigate these effects include advanced wastewater treatment technologies and the adoption of green chemistry solutions. This underscores the urgent need for coordinated global action to address the challenges posed by EDCs. These applications underscore MS’s pivotal role in understanding the distribution and impact of ECs on ecosystems and human health.

## 8. Recent Advances in MS for EC Detection

Recent advancements in MS have significantly enhanced the detection and analysis of ECs in environmental matrices. Innovations such as direct mass spectrometry techniques, including PTR-MS, have enabled the real-time monitoring of VOCs with high sensitivity and rapid response times. Salthammer et al. (2023) highlight the critical role of PTR-MS in analyzing organic compounds in indoor air environments [73]. This advanced technique provides the real-time monitoring of very volatile and volatile organic compounds (VVOCs and VOCs) with high sensitivity, often reaching detection limits in the parts-per-trillion (ppt) range. PTR-MS offers significant advantages over traditional chromatographic methods by enabling the direct analysis of complex mixtures in the gas phase. The authors utilized ion–dipole collision theories, such as Average Dipole Orientation and capture theory, to compute the proton transfer rate constants (*k*_PT_) for 114 organic compounds, supported by density functional theory (DFT) for the dipole moment and polarizability calculations. Key challenges identified include calibration difficulties, molecular fragmentation influenced by electric field strengths, and the importance of thermally averaged quantum chemical calculations to (*k*_PT_) accuracy. This work underscores PTR-MS as a versatile tool for applications in ECs detection.

Additionally, the integration of Ion Mobility Spectrometry-Mass Spectrometry (IMS-MS) has improved analytical selectivity by separating ions based on their shape and charge, thereby facilitating the differentiation of isomeric and isobaric species. Aly et al. demonstrated the utility of ion mobility spectrometry coupled with mass spectrometry (IMS-MS) for the rapid characterization and detection of persistent organic pollutants (POPs) and their metabolites [74]. This study evaluated [64] chemicals, including pesticides, industrial chemicals, pharmaceuticals, and polyfluoroalkyl substances (PFAS), using complementary ionization techniques like electrospray ionization (ESI) [75] and atmospheric pressure photoionization (APPI). IMS-MS allowed the simultaneous separation of parent compounds and their degradation products based on their molecular weight and drift time, enabling rapid screening without extensive sample preparation. Key findings include the superior sensitivity of ESI for polar metabolites and the ability of IMS-MS to distinguish isomers of hydroxylated and sulfated POPs. The results underscore the potential of IMS-MS as a robust screening tool for environmental pollutants, facilitating an exposure assessment and advancing the understanding of chemical degradation pathways.

**Figure 3:**
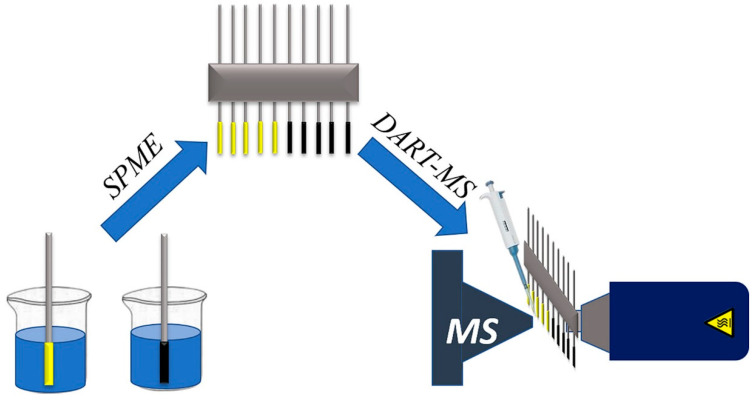
Scheme of SSE-DART-MS [77].

Ambient ionization techniques, such as Desorption Electrospray Ionization (DESI) and Direct Analysis in Real Time (DART), have revolutionized the field by allowing the direct sampling of surfaces under ambient conditions, thus reducing the sample preparation time and preserving the integrity of labile compounds. Raths et al. demonstrate the potential of Desorption Electrospray Ionization Mass Spectrometry (DESI-MS) in visualizing the spatial distribution of organic contaminants and their biotransformation products within amphipod tissues [76]. DESI-MS imaging, combined with cryosectioning, revealed high sensitivity for detecting small organic molecules, surpassing Matrix-Assisted Laser Desorption Ionization (MALDI-MS) in certain contexts. DESI’s minimal sample preparation and ability to detect compounds with lower fragmentation make it a valuable tool for environmental sciences, though advancements are needed to analyze samples at environmentally relevant concentrations. Jing et al. introduced a sorbent and solvent co-enhanced direct analysis in real-time mass spectrometry (SSE-DART-MS) method for the high-throughput detection of trace pollutants in water. By integrating graphitic carbon nitride (g-C_3_N_4_) sorbents with organic solvents, the technique achieved signal enhancements of up to 100-fold for phthalic acid esters (PAEs), with detection limits as low as 0.07 ng/L [77]. The approach combines the advantages of SPE and DART-MS, offering superior sensitivity and reduced matrix interference. This innovative method demonstrates potential for the rapid, environmentally friendly analysis of trace contaminants in complex samples (Figure 3).

High-Resolution Mass Spectrometry (HR-MS) has become a pivotal tool in identifying unknown ECs, offering precise mass measurements that aid in elucidating molecular formulas and structures. Giannelli Moneta et al. and Yang et al. emphasize the power of high-resolution mass spectrometry (HRMS) for the non-target screening of environmental contaminants. Giannelli Moneta et al. applied LC-HRMS to Arctic snow and aerosol samples, identifying over 150 compounds, including potential anthropogenic pollutants like plasticizers and flame retardants. This method highlighted the challenges of tracing sources due to limited overlap between snow and aerosol contaminants [78]. Yang et al. showcased HRMS’s utility in industrial settings, identifying new pollutants from metallurgical and incineration processes. They highlighted HRMS’s high sensitivity for ECs, enabling source identification and supporting sustainable industrial development [79]. Together, these studies underscore HRMS as a critical tool for tracking environmental pollutants and advancing sustainable practices. The application of HR-MS in non-target screening approaches has expanded the scope of environmental analysis, enabling the detection of previously unrecognized contaminants and their transformation products. These technological advancements collectively contribute to a more comprehensive understanding of ECs in the environment, enhancing the ability to monitor and mitigate their impact on ecosystems and human health.

## 9. Data Analysis and Interpretation in MS-Based EC Studies

Data analysis and interpretation in MS-based studies of ECs involve a multi-step process that is essential for extracting reliable and meaningful results from complex environmental samples. Advanced data processing techniques, including peak detection, spectral deconvolution, and an alignment of retention times, are fundamental to identifying contaminants amidst a high background of interfering substances [80]. These methods often rely on software tools and algorithms that can handle large, multidimensional datasets, ensuring the accurate quantification and identification of target and non-target compounds.

Bioinformatics and chemometric tools have become indispensable in analyzing MS data, especially in studies of ECs, which often involve complex mixtures. Techniques such as principal component analysis (PCA), hierarchical clustering, and machine learning models aid in identifying patterns, classifying contaminants, and correlating their presence to potential sources or environmental factors. For example, chemometric approaches can differentiate between anthropogenic and natural compounds, while machine learning can predict the behavior and degradation pathways of ECs based on MS data. These tools also facilitate the integration of MS data with other environmental datasets, offering a holistic understanding of contaminant behavior and impacts. Eysseric et al. presented a comprehensive workflow for the non-targeted screening (NTS) of trace organic contaminants in surface waters using high-resolution mass spectrometry (HRMS). Their approach integrated three complementary tools: Similar Partition Searching (SPS), Global Natural Products Social Networking (GNPS), and MetFrag for the analysis of tandem mass spectra. This combinatorial method enabled the identification of 253 contaminants, including pharmaceuticals, consumer product additives, and pesticides, with 44 compounds confirmed using reference standards [81]. Advanced computational techniques and empirical databases allowed the structural annotation and the detection of transformation products at ultra-trace levels. This study demonstrates the power of combining in silico tools and empirical data for robust, high-confidence contaminant analysis in complex environmental matrices. Meijer et al. developed the CECscreen database, an extensive resource for annotating chemicals of emerging concern (CECs) in non-targeted high-resolution mass spectrometry (HRMS) studies. This database aggregates over 70,000 “MS-ready” structures and includes simulated Phase I metabolites, expanding its applicability for exposome research. Advanced computational tools were used for structural standardization, physicochemical property prediction, and metabolite simulation, ensuring reliable annotations [82]. Integration with bioinformatics platforms like MetFrag facilitates chemical identification and prioritization based on environmental and toxicological relevance. This approach addresses challenges in the data analysis, offering a robust solution for identifying known and unknown CECs in complex biological matrices.

**Figure 4:**
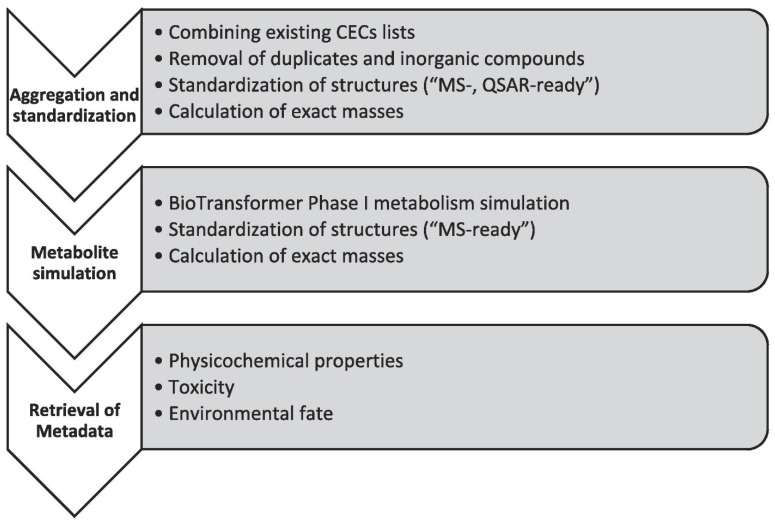
The three major steps and substeps in the pipeline to create an “MS-ready” database of CECs for screening in human samples. The first step is the aggregation of existing CECs lists and standardization to produce structure forms that can be measured with MS or are compliant with QSAR models. In the second step, metabolites are simulated, and their structures are standardized. In the final step, metadata are collected and predicted for physicochemical properties, environmental fate, and toxicity [82]. Reprinted with permission from Elsevier.

Figure 4 illustrates the three-step workflow for developing the CECscreen database for chemicals of emerging concern (CECs). The first step, aggregation and standardization, involves combining existing CEC lists, removing duplicates and inorganic compounds, standardizing structures into “MS-ready” and “QSAR-ready” formats, and calculating exact masses. The second step focuses on a metabolite simulation using a BioTransformer to predict Phase I metabolites and standardize their structures. The final step retrieves metadata, including physicochemical properties, toxicity, and environmental fate, to support the comprehensive chemical annotation in HRMS-based studies.

Despite these advances, several challenges hinder the interpretation of results and ensure reproducibility. Variations in sample preparation methods, instrumental settings, and data processing workflows can lead to inconsistencies between studies. Moreover, the lack of standardization in reporting MS data, including library matching criteria and ion fragmentation patterns, further complicates cross-study comparisons. Ensuring reproducibility requires the adoption of standardized protocols, rigorous quality control measures, and the development of open-access databases that allow researchers to share and validate their findings. Such databases can also serve as reference tools for the identification of unknown compounds, fostering collaboration and improving the reliability of MS-based EC studies.

## 10. Challenges in MS Analysis

### 10.1. Ionization of Complex Environmental Samples

For MS analysis, one significant challenge is the effective ionization of certain environmental samples, which may result in poor detection due to their inherent chemical properties. For instance, non-polar or thermally labile compounds often exhibit low ionization efficiency under conventional MS ionization techniques [83,84], such as Electrospray Ionization (ESI) or Atmospheric Pressure Chemical Ionization (APCI) [85]. In such cases, derivatization has emerged as a key strategy to enhance ionization by chemically modifying analytes to introduce functional groups that improve their ionization efficiency or stability [86].

Derivatization methods, such as silylation, acetylation, or methylation, have been widely employed to enable the detection of otherwise difficult-to-ionize compounds in environmental matrices [87]. For example, silylation is commonly used to detect hydroxyl-containing compounds like phenols and alcohols, whereas acetylation is effective for amines and carboxylic acids [88]. However, even with these advanced derivatization techniques, challenges persist. These include the need for precise reaction conditions, the potential introduction of artifacts, and additional sample preparation steps that may complicate the workflow and affect the reproducibility.

Recent advancements, such as the use of microfluidic systems for automated derivatization and the development of universal derivatization reagents, have shown promise in addressing these challenges [89]. However, the field continues to face limitations, particularly for non-targeted analyses where broad-spectrum applicability is required. Future research should focus on improving the derivatization efficiency, reducing reaction times, and developing reagents compatible with high-throughput analytical workflows.

### 10.2. High-Throughput Analysis of Environmental Samples

The MS analysis of environmental samples often involves enormous sample volumes and generates vast datasets due to the need for the comprehensive screening and quantification of diverse analytes [90,91]. This high throughput poses significant challenges in both the sample analysis and data processing. Analytical flux, which refers to the number of samples that can be analyzed within a given timeframe, is limited by factors such as the instrument speed, sample preparation complexity, and analytical run times [92]. For example, large-scale environmental monitoring studies require the analysis of hundreds to thousands of samples, straining traditional MS workflows [93].

In addition to throughput issues, the massive datasets generated by high-resolution MS and non-targeted analysis require sophisticated data processing tools and computational resources. Extracting meaningful information from such datasets is challenging due to the need for the peak deconvolution, alignment, and identification of unknown compounds. Furthermore, high data volumes increase the risk of missing critical information due to processing bottlenecks or manual review limitations.

Recent advancements in automation and data analysis offer promising solutions. Automated sample preparation systems and multiplexing techniques can significantly improve analytical flux by enabling the parallel processing of samples. For example, mixed-mode online SPE coupled with liquid chromatography–tandem mass spectrometry (LC-MS/MS) represents an advanced approach. This method integrates various sorbents to enhance the recovery of trace organic contaminants (TrOCs) from diverse water matrices, achieving method quantification limits often below 10 ng/L while reducing the solvent use and processing time [94]. On the data side, machine learning algorithms and cloud-based computational platforms are increasingly used to handle large datasets efficiently [95]. Automated sample preparation systems and multiplexing techniques can significantly improve the analytical flux by enabling the parallel processing of samples. For example, Zou et al. demonstrated the use of a novel mixed-mode online SPE coupled with LC-MS/MS to enhance the recovery of trace organic contaminants from environmental water matrices [94]. Similarly, Chen et al. introduced MS-PyCloud, a cloud-based pipeline for large-scale LC-MS/MS data analysis, which ensures scalability, transparency, and efficiency in processing proteomics datasets [96].

Future research should focus on integrating these advanced technologies into MS workflows to address the challenges posed by high sample and data volumes. Collaborative efforts between instrument developers, software engineers, and environmental scientists will be critical in ensuring that these solutions meet the demands of modern environmental monitoring.

## 11. Future Trends and Perspectives

Advancements in technology and digital tools are poised to reshape the role of MS in EC analysis, revolutionizing environmental monitoring. High-resolution and hybrid MS technologies, such as Orbitrap and time-of-flight (TOF) systems, are continually evolving, offering enhanced sensitivity, mass accuracy, and dynamic range for detecting trace-level contaminants in complex matrices. These advancements enable the improved non-targeted screening and identification of both known and unknown contaminants, facilitating comprehensive environmental surveillance [97,98]

The integration of artificial intelligence (AI) and machine learning into MS workflows is another transformative trend. These digital tools can automate peak identification, classify compounds, and predict transformation products based on large datasets, significantly reducing the manual data processing time while enhancing the accuracy. AI-driven algorithms are already being explored for pattern recognition, source apportionment, and the predictive modeling of contaminant behavior and fate in various environmental compartments [99].

Moreover, MS is emerging as a critical tool in predictive and preventive environmental monitoring, aiding in the early identification of pollutants and assessing their ecological and health risks. By enabling the detection of contaminants at ultra-trace levels and revealing transformation pathways, MS supports the development of mitigation strategies and policy interventions. For example, the real-time MS-based monitoring of the air and water quality can provide actionable data to prevent environmental crises, making it an invaluable tool for achieving sustainable environmental management goals [100].

These advancements underscore the role of MS in addressing the growing challenges posed by ECs, bridging the gap between analytical science and environmental protection, and supporting global efforts to safeguard ecosystems and public health.

## 12. Conclusions

ECs present significant environmental and health challenges due to their persistence, bioaccumulation potential, and often-unknown toxicological effects. Addressing these challenges requires robust analytical tools, with MS standing out as a pivotal technology for detecting, identifying, and quantifying ECs across diverse environmental matrices. The versatility of MS techniques, including GC-MS, LC-MS, PTR-MS, and HR-MS, has enabled unparalleled sensitivity and specificity, supporting the monitoring of contaminants at trace levels and advancing our understanding of their environmental behavior and impacts.

This review highlights the critical role of MS in environmental monitoring, from the real-time analysis of VOCs to the non-targeted identification of unknown pollutants. Despite its effectiveness, challenges such as matrix interferences, standardization gaps, and the need for comprehensive spectral libraries persist. These issues underline the importance of continued innovation in sample preparation methods, MS technologies, and data analysis approaches to improve the reliability and reproducibility of results. Looking forward, advancements in MS instrumentation, combined with the integration of artificial intelligence and machine learning, promise to revolutionize environmental monitoring. These technologies will enable more efficient data interpretation, the predictive modeling of contaminant behavior, and the development of targeted mitigation strategies. By fostering interdisciplinary collaboration and supporting regulatory efforts, MS will continue to play a vital role in addressing the complex issues posed by ECs, ensuring the protection of ecosystems and human health.

## Data Availability

No new data were created or analyzed in this study. Data sharing is not applicable to this article.
